# Do neutrophil extracellular traps implicate in atheromatous plaques from carotid endarterectomy? Re-analyzes of cDNA microarray data by surgeons

**DOI:** 10.3389/fneur.2023.1267136

**Published:** 2023-12-19

**Authors:** Ryotaro Takahira, Kenta Ujifuku, Tsuyoshi Izumo, Ang Xie, Kazuaki Okamura, Yoichi Morofuji, Takayuki Matsuo

**Affiliations:** Department of Neurosurgery, Graduate School of Biomedical Sciences, Nagasaki University, Nagasaki, Japan

**Keywords:** atheromatous plaque, carotid endarterectomy, cDNA microarray, neutrophil extracellular traps, peptidyl arginine deiminase 4

## Abstract

**Background:**

Carotid artery stenosis is the cause of 15% of strokes. Neutrophil extracellular traps (NETs) and peptidyl arginine deiminase 4 (PAD4) are believed to be involved in thrombosis. This pilot study described the differential expression profile of NETs between atheromatous plaques and surrounding tissues.

**Methods:**

Microarray datasets of carotid plaques were obtained from Gene Expression Omnibus. The normalized data were processed into comma-separated value matrix files using spreadsheet software. Analyzes of microarray data were conducted using integrated differential expression and pathway analysis.

**Result:**

The clustering results illustrated that the classifications of plaque and control had reasonable biological validity. Pathway analysis revealed the relevance of immune response, cell signaling, and other pathways. Differentially expressed genes were detected between carotid plaques and control specimens. However, enrichment analyzes did not reveal a difference in PAD4 expression between the groups and that NET implication was only found in one cDNA microarray dataset.

**Discussion:**

This pilot study does not necessarily dismiss the possibility of a relationship between NETs and atherothrombotic stroke. Gene expression could differ between endothelial cells and atheromas, and further studies are needed.

## 1 Introduction

Carotid artery stenosis is the cause of 15% of strokes (1, 2). Based on early histopathologic studies, ischemic events are associated with intraplaque hemorrhage, ulceration, calcification, lipid-rich necrosis, plaque thrombus, macrophage infiltration, and high microvessel density (3–6).

Neutrophil extracellular traps (NETs) are specialized structures released by neutrophils. NETs were initially believed to form in response to stimuli such as infection and inflammation and contribute to the elimination of pathogens such as bacteria and viruses (7). Recently, they have been suggested to participate in the regulation of inflammatory responses, blood coagulation, and pathological conditions such as autoimmune diseases and thrombosis (8–10). Elevated peptidyl arginine deiminase 4 (PAD4) levels have been detected in blood samples collected during carotid artery stenting, suggesting the involvement of NETs in the pathogenesis of atherothrombotic stroke (11).

Microarray and ribonucleic acid sequencing (RNAseq) allow comprehensive analyzes of transcriptomes. Genome-wide transcriptome analysis is often required in addition to individual gene expression analyzes. There are already re-analysis reports of existing microarray data (12, 13). However, big data analysis requires knowledge of statistics, informatics, and data science, which can pose difficulties for general biologists, physicians, and surgeons (14, 15).

In the absence of a bioinformatics expert, this study analyzed whether correlations related to NETs could be detected using historical carotid plaque-derived complementary deoxyribonucleic acid (cDNA) microarray data.

## 2 Materials and methods

Based on national ethical guidelines, this study did not originally fall under the category of research requiring written consent from study participants (16). This study was approved by the Institutional Review Board (number 23071016). The Gene Expression Omnibus^1^ database was examined using the search terms human, carotid artery, and endarterectomy. Twelve data were found as of October 2023. GSE28829 and GSE43292 datasets, which appeared to compare plaque and normal to early atheromatous vessels, were selected for the present analysis (Table 1) (17, 18). The downloaded normalized data were converted to comma-separated value (CSV) matrix files using spreadsheet software. An outline of the strategy used for the GEO original data is provided in the Supplementary Files S1–S4. Analyzes of microarray data were conducted using integrated Differential Expression and Pathway analysis (iDEP) 1.1 (19).^2^ The detailed methods and R session information are provided in the Supplementary File S5.

**Table 1:** Reanalyzed microarray data of carotid endarterectomy specimens.

Authors and Year	GEO accession number	Examined specimens, number	Comparison specimens, number	Array
Manca et al. (2011) (18)	GSE28829	Advanced lesion (thin or thick fibrous cap atheroma), 16	Early lesion (intimal thickening and intimal xanthoma), 13	Affymetrix Human Genome U133 Plus 2.0 Array
Bricca et al. (2013) (17)	GSE43292	Atheroma plaque (stage IV and over of the Stary classification) containing the core and shoulders of the plaque, 32	Distant macroscopically intact tissue (stages I and II), 32	Affymetrix Human Gene 1.0 ST Array [transcript (gene) version]

GEO, Gene expression omnibus. The normalized data files are downloaded from GEO (https://www.ncbi.nlm.nih.gov/geo/).

## 3 Results

### 3.1 Heatmap, principal component analysis, and differential expression analysis

**Figure 1:**
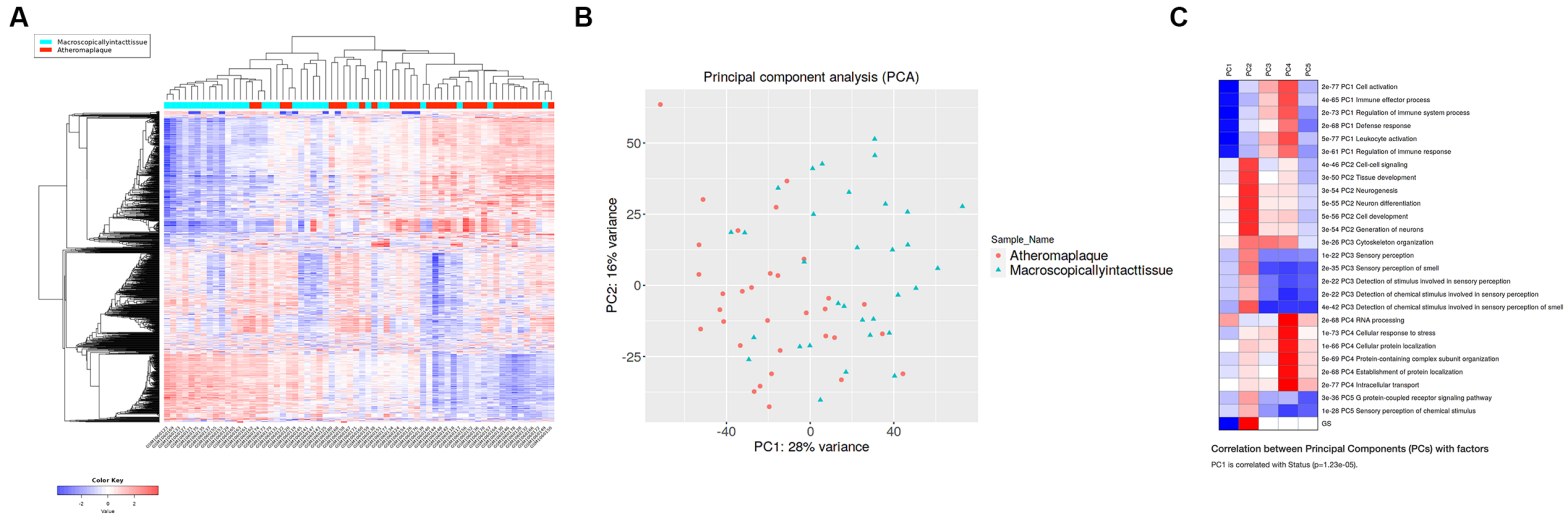
Hierarchical clustering heatmap **(A)** and principal component analysis **(B)** of advanced (unstable) and early (stable) atherosclerotic arteries from the GSE43292 dataset. The clusters are separated according to the pre-specified classification (plaque or normal), and the comparisons are likely to be meaningful. **(C)** GSE43292 pathway analysis of the PCA rotation matrix displays gene groups extracted using the results of principal component analysis. Inflammation, immune response, and other pathways were extracted.

**Figure 2:**
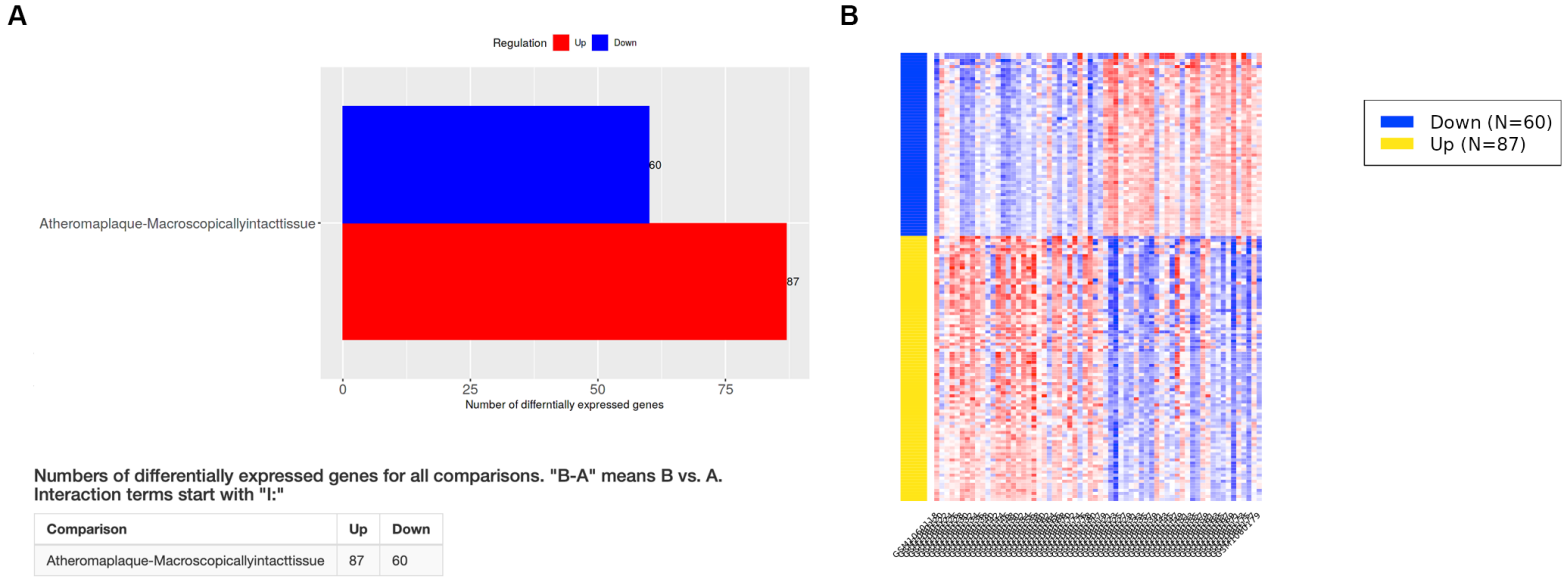
Differentially expressed genes (DEGs) in the GSE43292 dataset. **(A)** Compared to the findings for control specimens (microscopically normal artery), 87 upregulated and 60 downregulated DEGs were detected in advanced carotid plaque (*q* < 0.1). **(B)** Heatmap of DEGs in the GSE43292 dataset. See the Supplementary Files for detailed data (Supplementary Files S6, S7).

The elimination q-value (false discovery rate [FDR]) was 0.10 in the iDEP computation. The clustering results indicated that the pre-specified classification of plaque and control specimens had more than moderate biological validity (Figures 1A,B). In PCA, principal component 1 (PC1) was mainly relevant to immune response, and PC2 was related to cell signaling, tissue development, neurogenesis, and other pathways (Figure 1C; Supplementary Figure S1). Differentially expressed genes (DEGs) of advanced carotid plaque were detected. Compared to microscopically normal artery, 87 upregulated and 60 downregulated DEGs were detected in advanced carotid plaque in the GSE43292 dataset (*q* < 0.1; Figure 2). In comparison with early plaques, 396 upregulated and 71 downregulated genes were detected in advanced carotid plaque in the GSE28829 dataset (*q* < 0.1; Supplementary Figure S1). See the Supplementary Files for detailed specific genes (Supplementary Files S6, S8).

### 3.2 Enrichment and pathway analyzes

**Figure 3:**
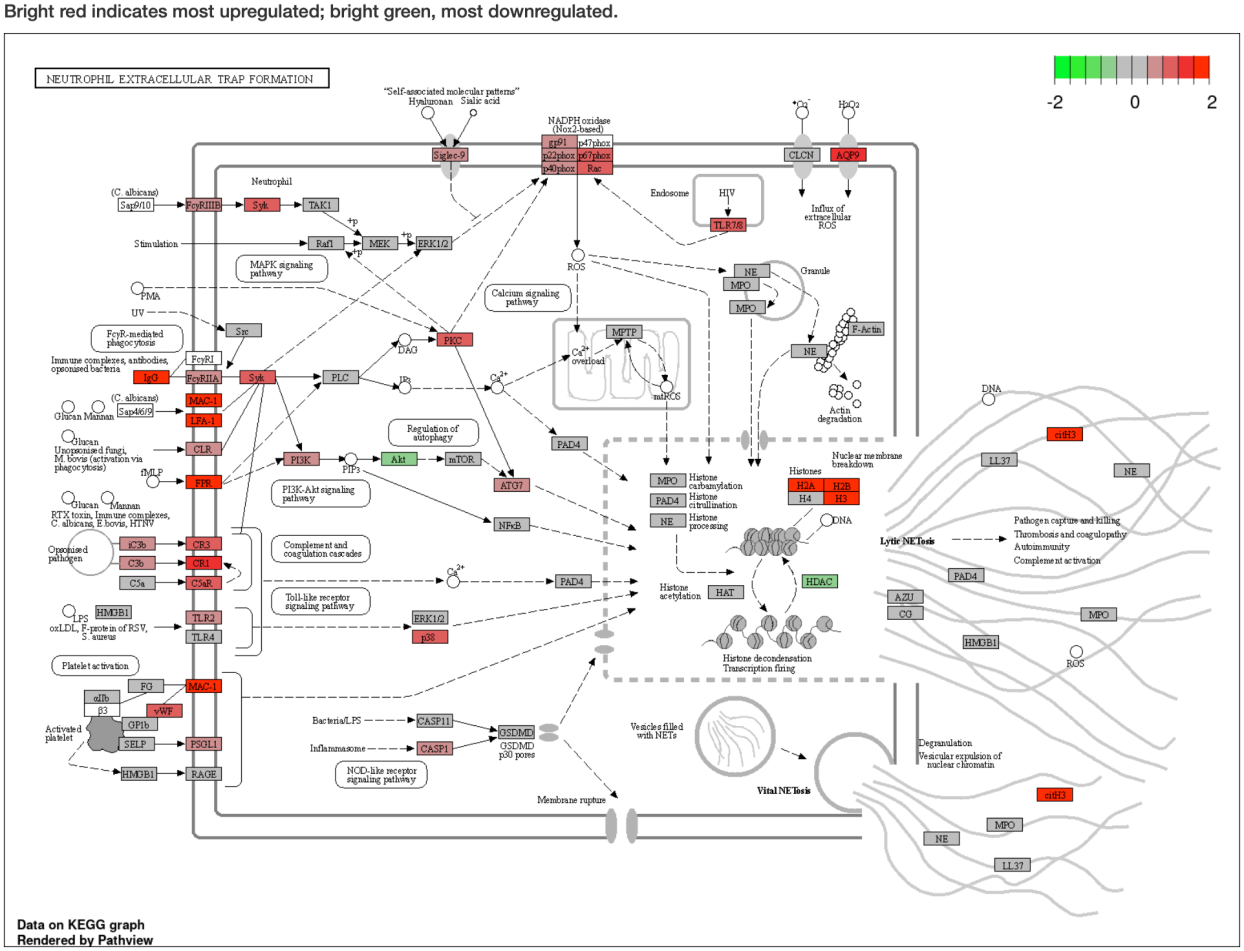
Pathway analysis of the GSE43292 dataset described in Kyoto Encyclopedia of Genes and Genomes graph. Neutrophil extracellular trap formation is enriched (false discovery rate < 0.2). Peptidyl arginine deiminase 4 (PAD4) expression was not significantly elevated. Histone deacetylase expression was reduced. Bright red indicates most upregulated; bright green, most downregulated. The KEGG pathway map (hsa04613 Neutrophil extracellular trap formation) is reprinted with permission from Kanehisa Laboratories.(20)

Pathway analysis was performed using Generally Applicable Gene-set Enrichment for Pathway Analysis (20) and Gene Ontology (21), and the selected gene sets were obtained from the Kyoto Encyclopedia of Genes and Genomes (KEGG) (22). The pathway significance cutoff (FDR) was 0.2. The main results are summarized in Table 2. The NET formation was enriched as a significant pathway only in the GSE43292 dataset, and DEGs were presented on the KEGG graph. PAD4 was not identified as a DEG in the expression analyzes. The two datasets shared the same reduced expression of histone deacetylase (HDAC), but differences were observed for histone expression (Figure 3; Supplementary Figure S1). See the Supplementary Files for detailed specific pathways (Supplementary Files S7, S9).

**Table 2:** Enriched pathways in both GSE28829 and GSE 43292 datasets.

Direction	Enriched pathway
Downregulated	Regulation of muscle contraction
Downregulated	Muscle contraction
Downregulated	Regulation of muscle system process
Upregulated	Immune response
Upregulated	Immune system process
Upregulated	Defense response
Upregulated	Response to other organism
Upregulated	Biological process involved in interspecies interaction between organisms
Upregulated	Inflammatory response
Upregulated	Cell activation

## 4 Discussion

Existing cDNA microarray data on carotid plaques of human origin represent a valuable source of information as they can be repeatedly analyzed to reflect the latest research, depending on the researcher’s interest. Nai et al. reported a re-analysis of the GSE43292 dataset and explored novel genes and pathways of carotid atheroma (12). Gao et al. examined immune cell infiltration between early and advanced carotid atheromatous plaque using the GSE28829 dataset (13). The cooperation of bioinformatics experts is considered essential for the former consideration. On the other hand, the latter report uses a web tool and does not necessarily require an expert, which could be another option from the present study. Our study presented a method for uploading normalized CSV matrix files to the iDEP web platform and analyzing the data (see legends in Supplementary Files S1–S4). All analyzes were performed on a graphical user interface such that the character user interface was avoided. As the analysis is performed online, a computer with standard performance was sufficient. Some typical cDNA microarray and RNAseq analysis methods are available and free of charge for scientific use (15, 19). We adopted this method in the present study because it allows visualization and display of the NETs’ DEG information on the KEGG graph.

The organization of the controls was not consistent in the present study (Table 1). Data from GSE28829 compared advanced plaque with intimal thickening and intimal xanthoma, and advanced plaque and distant macroscopically intact tissues were compared in GSE43292. One possible reason for the discrepancies between the results of the two datasets in this study could be that the former detected mainly DEGs associated with plaque progression, while the latter detected mainly DEGs associated with plaque development. The lack of control samples compared to the number of validation samples in the GSE28829 data may have also affected the results. Conversely, it remains nearly impossible to obtain human-derived normal arterial tissue as control samples from an ethical viewpoint.

High PAD4 expression was not extracted as a DEG in our re-analysis of existing microarray data. This finding is inconsistent with that reported by Simonaga et al. (11). They collected blood samples from the luminal side, which could represent a different target from our study results, in which atheromas were analyzed. In other words, it is possible that different genes could be expressed in vascular endothelial cells and atheromas even though both contribute to a series of atherosclerotic processes. Therefore, we cannot exclude the possibility that NETs are involved in the development of carotid artery plaques and their rupture. Clinicopathological studies and single-cell comprehensive gene expression analyzes could be helpful for clarifying their pathogenesis.

Several limitations to this study warrant mention. Because of the inconsistency of the controls, whether they represented normal tissue may be debatable (Table 1). Next, microarrays are not chip-compatible, making integrated analysis extremely difficult. Then, although the results of analyzes of cDNA microarray and RNAseq data can suggest certain correlations, causal relationships cannot always be proven. Furthermore, scientists should consider the final biological interpretation as the results of big data and machine learning do not necessarily have biological relevance (15, 23). Finally, this research is an analysis that is only possible within the platform created by bioinformatics researchers. The need to rely on experts will continue to be necessary when detailed fine-tuning or new analysis methods are required.

## Data availability statement

The original contributions presented in the study are included in the article/Supplementary material, further inquiries can be directed to the corresponding author.

## Ethics statement

The studies involving humans were approved by the Nagasaki University Hospital Institutional Review Board. The studies were conducted in accordance with the local legislation and institutional requirements. The human samples used in this study were acquired from another research group. Written informed consent for participation was not required from the participants or the participants’ legal guardians/next of kin in accordance with the national legislation and institutional requirements.

## Author contributions

RT: Conceptualization, Data curation, Formal analysis, Methodology, Writing – original draft, Writing – review & editing. KU: Conceptualization, Data curation, Formal analysis, Funding acquisition, Methodology, Project administration, Resources, Software, Writing – original draft, Writing – review & editing. TI: Conceptualization, Formal analysis, Funding acquisition, Investigation, Project administration, Supervision, Validation, Visualization, Writing – original draft, Writing – review & editing. AX: Data curation, Writing – review & editing. KO: Writing – review & editing. YM: Funding acquisition, Writing – review & editing. TM: Funding acquisition, Supervision, Writing – review & editing.

## Footnotes

^1^GEO, https://www.ncbi.nlm.nih.gov/geo/.

^2^ http://ge-lab.org/idep/

## Supplementary material

Supplementary Datasheet 1-4 Data for integrated Differential Expression and Pathway analysis. Matrix data were processed using spreadsheet software and saved as CSV files (Suppl1GSE28829_series_matrix, and Suppl3GSE43292_series_matrix). Attribute data were additionally attached (Suppl2GSE28829_series_attribute, and Suppl4GSE43292_series_attribute). When you process original Gene Expression Omnibus data, (1) the 1 × 1 cell must be blank data. (2) Paste the gene name or ID column from the 2 × 1 cell. Select the column, and specify the display format as a character string. (3) Provide the names of the specimens from the 1 × 2 cell. (4) Paste the expression data and complete the matrix data. (5) Remove the unnecessary description of statistics and other data. Unnecessary cells, columns, and rows should be removed. (6) Save the matrix as a CSV file. (15) See the Suppl1GSE28829_series_matrix and Suppl3GSE43292_series_matrix files for examples.

Supplementary Datasheet 5 GSE43292 differentially expressed genes.

Supplementary Datasheet 6 GSE43292 enriched phenomena.

Supplementary File 7 GSE28829 differentially expressed genes.

Supplementary File 8 GSE28829 enriched phenomena.
