# Supplementary material for: Do neutrophil extracellular traps implicate in atheromatous plaques from carotid endarterectomy? Re-analyzes of cDNA microarray data by surgeons
Source: Front Neurol. 2023 Dec 19;14:1267136. doi: 10.3389/fneur.2023.1267136 (PMC10770953; doi:10.3389/fneur.2023.1267136)
Supplement: Supplementary file 6 [file Table_1.DOCX]

**Supplementary file 1.** Log of integrated Differentially Expression and Pathway.

***Data***Species: Human genes (GRCh38.p13)
Number of samples: 29 (GSE28829) and 64 (GSE43292), respectively.
Number of genes converted and filtered: 54675 (GSE28829) and 33297 (GSE43292), respectively.
2 sample groups.
Input file type: normalized expression file

***Pre-processing and exploratory data analysis settings***
Low filter: lowFilter= -1000
No log-transformation.
number of genes in heatmap: nGenes= 1000
number of genes in k-means clustering: nGenesKNN= 2000
number of clusters in k-means clustering: nClusters= 4
Promoter analysis for k-means clustering: radioPromoterKmeans= 300 bp

***Differential expression settings***
FDR cutoff: limmaPval= 0.1
Fold-change cutoff: limmaFC= 2
Promoter analysis for DEGs: radio.promoter= 300 bp

***Pathway analysis settings***
Pathway analysis methods: pathwayMethod= GAGE
FDR cutoff: pathwayPvalCutoff= 0.1
Min size for gene set: minSetSize= 5
Max size for gene set: maxSetSize= 2000

**Supplementary R session information**

R version 4.2.1 (2022-06-23)
Platform: x86_64-pc-linux-gnu (64-bit)
Running under: Ubuntu 20.04.5 LTS

Matrix products: default
BLAS: /usr/lib/x86_64-linux-gnu/openblas-pthread/libblas.so.3
LAPACK: /usr/lib/x86_64-linux-gnu/openblas-pthread/liblapack.so.3

locale:
[1] LC_CTYPE=en_US.UTF-8 LC_NUMERIC=C  
[3] LC_TIME=en_US.UTF-8 LC_COLLATE=en_US.UTF-8  
[5] LC_MONETARY=en_US.UTF-8 LC_MESSAGES=en_US.UTF-8  
[7] LC_PAPER=en_US.UTF-8 LC_NAME=C  
[9] LC_ADDRESS=C LC_TELEPHONE=C  
[11] LC_MEASUREMENT=en_US.UTF-8 LC_IDENTIFICATION=C  

attached base packages:
[1] stats4 stats graphics grDevices utils datasets methods  
[8] base  

other attached packages:
[1] pathview_1.36.1 gage_2.46.1  
[3] statmod_1.4.37 PGSEA_1.60.0  
[5] annaffy_1.68.0 BiocManager_1.30.18  
[7] KEGG.db_2.8.0 GO.db_3.15.0  
[9] AnnotationDbi_1.58.0 DESeq2_1.36.0  
[11] SummarizedExperiment_1.26.1 Biobase_2.56.0  
[13] MatrixGenerics_1.8.1 matrixStats_0.62.0  
[15] GenomicRanges_1.48.0 GenomeInfoDb_1.32.4  
[17] IRanges_2.30.1 S4Vectors_0.34.0  
[19] BiocGenerics_0.42.0 edgeR_3.38.4  
[21] limma_3.52.4 reshape2_1.4.4  
[23] DT_0.25 e1071_1.7-11  
[25] gplots_3.1.3 RSQLite_2.2.17  
[27] visNetwork_2.1.2 reactable_0.3.0  
[29] shinyjs_2.1.0 plotly_4.10.0  
[31] ggplot2_3.3.6 shinyBS_0.61.1  
[33] shinyAce_0.4.2 shiny_1.7.2  

loaded via a namespace (and not attached):
[1] colorspace_2.0-3 ellipsis_0.3.2 class_7.3-20  
[4] XVector_0.36.0 proxy_0.4-27 farver_2.1.1  
[7] bit64_4.0.5 fansi_1.0.3 codetools_0.2-18  
[10] splines_4.2.1 cachem_1.0.6 geneplotter_1.74.0  
[13] jsonlite_1.8.0 annotate_1.74.0 png_0.1-7  
[16] graph_1.74.0 compiler_4.2.1 httr_1.4.4  
[19] assertthat_0.2.1 Matrix_1.5-1 fastmap_1.1.0  
[22] lazyeval_0.2.2 cli_3.4.1 later_1.3.0  
[25] htmltools_0.5.3 tools_4.2.1 gtable_0.3.1  
[28] glue_1.6.2 GenomeInfoDbData_1.2.8 dplyr_1.0.10  
[31] Rcpp_1.0.9 jquerylib_0.1.4 vctrs_0.4.2  
[34] Biostrings_2.64.1 crosstalk_1.2.0 stringr_1.4.1  
[37] mime_0.12 lifecycle_1.0.2 gtools_3.9.3  
[40] XML_3.99-0.10 org.Hs.eg.db_3.15.0 zlibbioc_1.42.0  
[43] scales_1.2.1 ragg_1.2.3 promises_1.2.0.1  
[46] KEGGgraph_1.56.0 parallel_4.2.1 RColorBrewer_1.1-3  
[49] yaml_2.3.5 memoise_2.0.1 sass_0.4.2  
[52] stringi_1.7.8 genefilter_1.78.0 caTools_1.18.2  
[55] BiocParallel_1.30.3 rlang_1.0.6 pkgconfig_2.0.3  
[58] systemfonts_1.0.4 bitops_1.0-7 lattice_0.20-45  
[61] fontawesome_0.3.0 purrr_0.3.4 htmlwidgets_1.5.4  
[64] labeling_0.4.2 bit_4.0.4 tidyselect_1.1.2  
[67] plyr_1.8.7 magrittr_2.0.3 R6_2.5.1  
[70] generics_0.1.3 DelayedArray_0.22.0 DBI_1.1.3  
[73] pillar_1.8.1 withr_2.5.0 survival_3.4-0  
[76] KEGGREST_1.36.3 RCurl_1.98-1.8 tibble_3.1.8  
[79] crayon_1.5.2 KernSmooth_2.23-20 utf8_1.2.2  
[82] locfit_1.5-9.6 grid_4.2.1 data.table_1.14.2  
[85] Rgraphviz_2.40.0 blob_1.2.3 digest_0.6.29  
[88] xtable_1.8-4 tidyr_1.2.1 httpuv_1.6.6  
[91] textshaping_0.3.6 munsell_0.5.0 viridisLite_0.4.1  
[94] bslib_0.4.0
