# Supplementary figures and images for: Do neutrophil extracellular traps implicate in atheromatous plaques from carotid endarterectomy? Re-analyzes of cDNA microarray data by surgeons
Source: Front Neurol. 2023 Dec 19;14:1267136. doi: 10.3389/fneur.2023.1267136 (PMC10770953; doi:10.3389/fneur.2023.1267136)

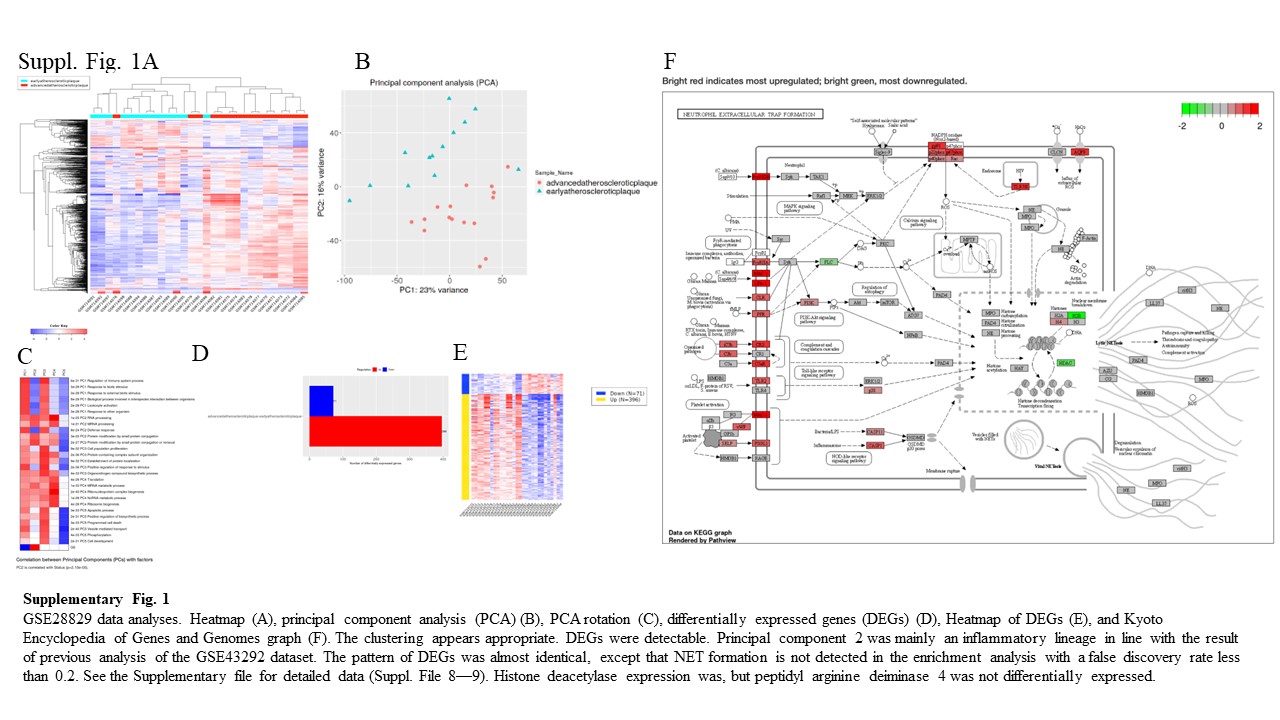

Supplement: Supplementary file 10 [file Image_1.jpg]
